# Efficacy and safety of 12 versus 48 months of dual antiplatelet therapy after implantation of a drug-eluting stent: the OPTImal DUAL antiplatelet therapy (OPTIDUAL) trial: study protocol for a randomized controlled trial

**DOI:** 10.1186/1745-6215-14-56

**Published:** 2013-02-21

**Authors:** Gérard Helft, Claude Le Feuvre, Jean Louis Georges, Didier Carrie, Florence Leclercq, Hélène Eltchaninoff, Alain Furber, Fabrice Prunier, Laurent Sebagh, Simon Cattan, Guillaume Cayla, Eric Vicaut, Jean-Philippe Metzger

**Affiliations:** 1 bd Vincent Auriol, Institut de Cardiologie, Hôpital Pitié-Salpétrière, Paris, France; 2 rue de Versailles Hôpital Mignot, Versailles, France; 3 avenue Jean Poulhes, CHU, Toulouse, France; 4 avenue du Doyen Gaston Giraud Hôpital CHU, Montpellier, France; 5 rue de Germont, CHU, Rouen, France; 6 rue Larrey, CHU, Angers, France; 7 rue de Turin Clinique Turin, Paris, France; 8 10, rue du Général Leclerc, Hôpital Le Raincy, Le Raincy, France; 9 place du Pr-Debré GHU, Carémeau, Nîmes, France; 10 rue du Faubourg St Denis URC Hôpital, Lariboisière, Paris, France

**Keywords:** Drug-eluting stent, Clopidogrel, Coronary artery disease, Stent thrombosis, Randomized clinical trial

## Abstract

**Background:**

Dual antiplatelet therapy with aspirin and thienopyridine is required after placement of coronary drug-eluting stents (DES) to prevent thrombotic complications. Current clinical guidelines recommend at least 6 to 12 months of treatment after a DES implantation, but it may be beneficial to apply dual antiplatelet therapy for a longer duration.

**Methods/design:**

The optimal dual antiplatelet therapy (OPTIDUAL) study aims to compare the benefits and risks of dual antiplatelet therapy applied for either 12 or 48 months. We will examine the occurrence of major adverse cardiovascular and cerebrovascular events (MACCE) in patients undergoing percutaneous coronary intervention with DES for the treatment of coronary lesions. The OPTIDUAL study is an open-label multicenter, randomized, national trial that will include 1,966 patients treated with DES. All patients will be treated with dual antiplatelet therapy for 12 months (+/− 3). Then, patients with no MACCE or major bleeding will be randomized to receive either 36 additional months of clopidogrel plus aspirin or aspirin only. The primary end-point is the combination of death from all causes, myocardial infarction, stroke and major bleeding. The secondary end points include the individual components of the primary end-point, stent thrombosis, repeat revascularization of the treated vessel and minor bleeding.

**Discussion:**

This randomized trial is designed to assess the benefits and safety of 12 versus 48 months of dual antiplatelet therapy in patients that receive a DES. We aim to determine whether substantial prolongation of clopidogrel (a thienopyridine) after DES implantation offers an advantage over its discontinuation.

**Trial registration:**

ClinicalTrials.gov Identifier: NCT00822536

## Background

In the first series of patients receiving a bare metal stent (BMS), stent thrombosis was already recognized as a severe complication after implantation owing to its high mortality. With the introduction of P2Y-receptor antagonists (that is, ticlopidine, clopidogrel) for platelet inhibition in combination with acetylsalicylic acid (ASA), the incidence of stent thrombosis decreased substantially in stable patients to levels as low as 1% [1]. Randomized controlled trials have established the efficacy of clopidogrel therapy following hospitalization in patients with acute coronary syndrome (ACS) that were treated either medically or with percutaneous coronary intervention (PCI) [2-4]. The implantation of drug-eluting stents (DES) has become a standard treatment for the management of patients with coronary artery disease. The widespread use of DES has significantly reduced the risk for in-stent restenosis compared to BMS [5,6]. However, there is some concern that DES might be associated with high rates of stent thrombosis, particularly beyond the first year after implantation [7]. The dominant risk factor for the occurrence of late stent thrombosis is discontinuation of antiplatelet therapy, including aspirin and thienopyridines (clopidogrel and prasugrel). Late stent thrombosis occurs within the first year after implantation of a DES [8-13]. It remains unknown whether it might be advantageous to prolong the 12-month antiplatelet thienopyridine (clopidogrel) therapy after DES implantation.

A recent update of the guidelines for PCI from the American College of Cardiology/American Heart Association/Society of Cardiovascular Angiography and Interventions stressed that all patients that receive a DES should be given clopidogrel treatment for at least 12 months in the absence of increased risk of bleeding [14]. Interestingly, the European Society of Cardiology guidelines on PCI differ slightly; they recommend clopidogrel therapy for 6 to 12 months after implantation of a DES, and for 9 to 12 months when a stenting procedure follows a presentation of an acute coronary syndrome (ACS) [15].

Early observational studies have suggested that extended use of clopidogrel in patients with a DES may be associated with reduced risk of death and myocardial infarction (MI) [7]. Additional studies have observed a clustering of adverse events in the initial 90 days after discontinuation of clopidogrel; this suggested the possibility of a clopidogrel rebound effect [16]. Based on those results, and also taking into account results from the Clopidogrel versus Aspirin in Patients at Risk of Ischemic Events (CAPRIE) study, some experts have advocated indefinite clopidogrel therapy [17]. However, when considering the potential beneficial effects of dual antiplatelet therapy, it is important to be aware that extending dual antiplatelet therapy beyond one year may induce bleeding complications. A meta-analysis of dual antiplatelet therapy has shown that this combination, compared to aspirin alone, reduced the risk of death, reinfarction and stroke by 15 to 34%; however, this benefit occurred at the expense of excess bleeding (odds ratio 1.80, 95% confidence interval 1.40 to 2.30) [18].

Very recently, three trials have been published suggesting that a shorter duration of dual antiplatelet therapy after stent implantation may be beneficial [19-21]. However, to date, it is unknown whether prolonged dual antiplatelet therapy will decrease major adverse cardiovascular and cerebrovascular events (MACCE) that occur more than 12 months after stent implantation, including very late stent thrombosis. Furthermore, it is uncertain what the optimal duration would be for long-term dual antiplatelet therapy. Therefore, given the lack of data from randomized studies, the aim of the OPTIDUAL study is to assess the benefits and safety of 12 versus 48 months of dual antiplatelet therapy in patients that receive a DES.

## Methods/design

The primary objective of the OPTIDUAL study (URL: ClinicalTrials.gov, number NCT00822536; approved by the ethical committee, number CPP 34–2008) is to compare the efficacy of 12 versus 48 months of dual antiplatelet therapy after DES implantation. We will test the hypothesis that 48 months is superior to 12 months of dual antiplatelet therapy.

### Population

The study population will consist of 1,966 patients that have been taking dual antiplatelet therapy for 12 months (+/− 3) after DES implantation. All patients will provide written informed consent to participate. At the beginning of the OPTIDUAL study, the patients will be randomized to either discontinue clopidogrel (12 months total) or to receive clopidogrel for an additional 36 months (48 months total). Inclusion and exclusion criteria are detailed in Table 1. No exclusion criterion is based on the type of DES implanted. Patients will be randomized without regard to the clinical status at presentation, including ACS, angina and silent myocardial ischemia. All enrolled subjects will have been treated with an approved DES and will have received 12 months (+/− 3) of aspirin plus thienopyridine. Doses of aspirin and clopidogrel will be based on the local standard of practice. Subjects may be enrolled into the study starting from immediately after the DES implantation, up to 12 months (+/− 3) after the DES, provided that they are on dual antiplatelet therapy. Dual antiplatelet therapy must include at least six months of aspirin plus clopidogrel (three months of initial prasugrel is permitted). A total of 1,966 patients will be randomized at 40 clinical study sites in France.

**Table 1:** Inclusion and exclusion criteria

Inclusion criteria:	1. Patients with DES implanted, then treated with clopidogrel plus aspirin for 12 months
	2. Informed, written consent from the patient
Exclusion criteria:	1. Age <18 years
	2. Oral anticoagulation therapy
	3. Drug-eluting stent in an unprotected left main coronary artery
	4. Contemporaneous enrollment in a different clinical trial
	5. Malignancies or other comorbid conditions with a life expectancy <2 years
	6. Known allergy or intolerance to the study medications: aspirin and/or clopidogrel
	7. Other revascularization with a DES within nine months prior to this study
	8. Other revascularization with a BMS within four weeks prior to this study

### Study design

#### Inclusion/exclusion criteria

The major exclusion criteria include 1) taking an oral anticoagulant therapy and 2) DES implantation in an unprotected left main coronary artery. BMS implantation in the same procedure is allowed. The complete list of inclusion and exclusion criteria is provided in Table 1. Malignancies and other comorbid conditions with a life expectancy <2 years are exclusion criteria screened by the principal investigator of each site. Patients with DES treated for 12 months (+/− 3) with aspirin and clopidogrel and who are event-free since the stent implantation (from myocardial infarction (MI), stent thrombosis, acute coronary syndrome, stroke, repeat coronary revascularization with a DES in the nine previous months or with a BMS in the last four weeks, moderate or major bleeding) are eligible for randomization.

### Randomization

The patients are randomized (1:1) to receive either aspirin alone or a continuation of clopidogrel plus aspirin for an additional 36 months. Randomization is stratified by centers. A centralized telephone randomization will be used with an interactive voice response system that operates 24 h per day, seven days per week. This system will provide the investigator with a subject number and treatment arm.

### Dosing regimens

After the index procedure, all subjects will take a daily dose of open-label clopidogrel and aspirin for 9 to 15 months after the index procedure. To minimize bleeding risk, the study will recommend that the aspirin dose should be the lowest acceptable dose, based on the physician’s discretion (75 to 160 mg) [22]. The recommended clopidogrel dosing will be a loading dose between 300 and 600 mg, and a maintenance dose of 75 mg daily to be continued for the duration of the treatment. Compliance is checked by a patient’s diary where the daily dose of each drug is reported. According to the results of CURENT-OASIS-7, clopidogrel dosing could be 150 mg per day for one week after the stent implantation in the setting of ACS [23]. Prasugrel could be administered after a PCI, but for no longer than three months. Patients that are not switched from prasugrel to clopidogrel at three months will not be randomized.

### Follow-up

All randomized patients will be followed up for 36 months after randomization. Thus, patients randomized in the clopidogrel group will receive dual antiplatelet therapy (clopidogrel plus aspirin) for a total of four years (+/− three months). The other patients will receive aspirin alone (75 to 160 mg daily). Demographic, clinical and procedural information will be captured at the time of enrollment. In addition, we will record subsequent clinical end-points, serious adverse events and concomitant medications. Time of randomization that is the time when the studied strategy is beginning (that is, stopped or sustained dual therapy) will be considered as the time 0 for study endpoints. Subjects will be contacted at 6, 12, 18, 24, 30 and 36 months after the randomization; that is, up to 48 months after the DES implantation. All end points and events that occur after randomization will be adjudicated by an independent Clinical Events committee, blinded to the assignment strategy. The committee will comprise physicians that are provided with all the data from medical records necessary to perform optimal adjudication.

### Study end-points

The primary end-point of the study is the incidence of a composite end-point, including all-cause mortality, MI, stroke and major bleeding events. The MIs will be classified and adjudicated according to the Academic Research Consortium (ARC) definition [24]. Major bleeding events will be classified as fatal bleeding, intra-cranial bleeding (assessed by MRI, CT, or autopsy), hemorrhagic shock, a need for transfusion of at least two units of packed red blood cells, reductions in hemoglobin greater than 3 g/dl, intra-ocular bleeding that leads to visual loss or bleeding that requires surgery.

The secondary end-points of the study include the individual components of the primary composite end-point, stent thrombosis according to the ARC definition, repeat vessel revascularization and minor bleeding events.

### Statistical considerations

#### Sample size

A sample size of 983 patients per group was calculated to provide an 80% power to demonstrate a difference between the two groups by survival analysis based on a Cox model. We assumed that the primary composite endpoint within three years post-randomization would be equal to 7% in one group vs. 4% in the other with an accrual period of 36 months. We also assumed a 5% two-sided significance level [25].

#### Study termination rules

An interim analysis will be made after 50% of the expected events have occurred. The study can be stopped to evaluate efficacy based on the conservative criterion of Peto (*P* <0.001). Based on a Bayesian calculation of conditional power (with Addplan software GmbH Koln Germany), the sample size can be reassessed and, when deemed futile, the study can be terminated [26].

#### Statistical analysis

All analyses will include the intention-to-treat population (all patients randomly assigned to treatment groups and analyzed as randomized, that is, patients crossing-over from one strategy to another will be analyzed in function of their group of randomization). Patients who drop-out will be censored at the time of the last available information.

The primary and secondary endpoints that are related to the time to an event will be analyzed with a survival analysis based on a Cox model. Alternative statistical models will be used when hypotheses regarding risks cannot be suitably analyzed with the Cox model. Other secondary endpoints will be analyzed with the *χ*^2^ or Fisher’s exact test for frequent comparisons. Two-sided significance level is fixed at 5%. All tests will be performed with SAS version 9.3 (SAS Institute Inc., Cary, NorthCarolina, USA).

### Study management

The OPTIDUAL study is planned to be a multicenter open-label trial, conducted in France, and sponsored by the Assistance Publique des Hôpitaux de Paris (Paris, France). Funding has been obtained from the Assistance Publique des Hôpitaux de Paris. An executive committee composed of experienced clinical investigators will provide trial leadership. A clinical events committee, blinded to the assignment strategy, will adjudicate all clinical events and a Data and Safety Monitoring Board (DSMB) is working. The committee will comprise physicians that are provided with all the data from medical records necessary to perform optimal adjudication.

## Discussion

Several pivotal clinical trials have shown that, compared to a BMS, the DES is associated with significant reductions in the risk of restenosis and the need for target-lesion revascularization [5,6]. Early discontinuation of dual antiplatelet therapy has been identified as a risk factor for late stent thrombosis in patients with DES [9]. Furthermore, it was suggested that some late clinical events that occur later than one year after DES implantation may be due to delayed arterial healing after the implantation of DES. Therefore, current guidelines recommend aspirin and clopidogrel at a dose of 75 mg daily for at least 12 months after DES implantation for patients that are not at high risk for bleeding. However, it remains unknown what the optimal duration might be for dual antiplatelet therapy and whether the risk-benefit ratio could be improved with long-term dual antiplatelet therapy for patients that receive DES. Due to the large number of DES implantations in the world each year, the optimization of dual antiplatelet therapy is important for both patient recovery and economic efficiency. The first randomized study on this issue was performed in Korea [19]. They analyzed combined data from two multicenter trials, the REAL-LATE and the ZEST-LATE trials. That study found that no significant benefit was associated with continuing clopidogrel plus aspirin beyond the 12-month treatment following DES implantation. They saw no reductions in the incidence of myocardial infarction or death from cardiac causes. Moreover, the rates of composite outcomes (MI, stroke, death) were higher with clopidogrel plus aspirin than with aspirin alone, although the difference was not significant. Recently, two additional studies investigated the effects of long-term dual antiplatelet therapy. The EXCELLENT study, by Gwon *et al*., showed that, at 12 months after DES implantation, patients treated with 12-month and 6-month dual antiplatelet therapy showed similar risks for target vessel failure, defined as the composite of cardiac death, MI and ischemia-driven target vessel revascularization [20]. However, the non-inferiority margin was wide, and the study was underpowered for death and MI. In the other study, by Valgimigli *et al*., 24 months of clopidogrel therapy in patients with DES or BMS was not significantly more effective than a 6-month clopidogrel regimen for reducing the composite endpoint of death, MI and cerebrovascular accident [21]. In that study, two years of clopidogrel therapy resulted in a significant increase in bleeding episodes. Although those studies were interesting, and they suggested the possibility that a shorter dual antiplatelet therapy might be sufficient after DES implantation, they did not rule out the possibility that prolonging the clopidogrel plus aspirin treatment might result in a reduction of MACCE.

Given the importance of this subject, it is not surprising that a few clinical trials are currently ongoing to investigate optimal treatment times. The dual antiplatelet therapy (DAPT) study aims to compare the benefits and risks of 12 versus 30 months of dual antiplatelet therapy in patients undergoing PCI, and it has the largest number of subjects [27]. Over 20,000 subjects will be enrolled at approximately 220 international clinical study sites. Most of the patients (>15,000) will receive a DES. Interestingly, in that prolonged combined antiplatelet therapy, patients will receive either clopidogrel or prasugrel, a new thienopyridine that recently appeared on the market. Another interesting ongoing trial aims to assess whether discontinuation of clopidogrel plus aspirin at six months after DES implantation would be non-inferior to a routine, one-year treatment [28].

## Conclusion

Our trial will conduct a substantially prolonged treatment of clopidogrel plus aspirin, for up to four years after DES implantation. We aim to determine whether long-term treatment would be superior to discontinuation at one year.

## Trial status

This study is currently recruiting patients.

## Abbreviations

ACS: Acute coronary syndrome;ARC: Academic research consortium;ASA: Acetylsalicylic acid;BMS: Bare metal stent;CAPRIE: Clopidogrel versus aspirin in patients at risk of ischemic events;DAPT: Dual antiplatelet therapy;DES: Drug-eluting stents;DSMB: Data and safety monitoring board;MACCE: Major adverse cardiovascular and cerebrovascular events;MI: Myocardial infarction;OPTIDUAL: Optimal dual antiplatelet therapy;PCI: Percutaneous coronary intervention

## Competing interests

This study is funded by the AP-HP (PHRC) and grants from Cordis, Boston, Medtronic, Terumo and Biotronik.

## Authors’ contributions

GH, CLF and JPM conceived the study. GH, CLF, JPM and EV participated in the study design. GH, CLF, JLG, DC, FL, HE, AF, FP, LS, SC and GC will recruit, select and collect clinical data of the patients. EV will perform statistical analysis. GH is the principal investigator. GH and EV drafted the manuscript. All authors read and approved the final manuscript.
